# LDL-Migration Index (LDL-MI), an Indicator of Small Dense Low-Density Lipoprotein (sdLDL), Is Higher in Non-Alcoholic Steatohepatitis than in Non-Alcoholic Fatty Liver: A Multicenter Cross-Sectional Study

**DOI:** 10.1371/journal.pone.0115403

**Published:** 2014-12-26

**Authors:** Kento Imajo, Hideyuki Hyogo, Masato Yoneda, Yasushi Honda, Takaomi Kessoku, Wataru Tomeno, Yuji Ogawa, Masataka Taguri, Hironori Mawatari, Yuichi Nozaki, Koji Fujita, Hiroyuki Kirikoshi, Satoru Saito, Yoshio Sumida, Masafumi Ono, Koichiro Wada, Atsushi Nakajima, Yuichiro Eguchi

**Affiliations:** 1 Department of Gastroenterology, Yokohama City University Graduate School of Medicine, Yokohama, Japan; 2 Department of Medicine and Molecular Science, Graduate School of Biomedical Sciences, Hiroshima University, Hiroshima, Japan; 3 Department of Biostatistics and Epidemiology, Yokohama City University Graduate School of Medicine, Yokohama, Japan; 4 Department of Gastroenterology and Hepatology, Kyoto Prefectural University of Medicine, Kamigyo-ku, Kyoto, Japan; 5 Department of Gastroenterology and Hepatology, Kochi Medical School, Kochi, Japan; 6 Department of Pharmacology, Osaka University Graduate School of Dentistry, Osaka, Japan; 7 Division of Hepatology, Saga Medical School, Liver Center, Saga, Japan; Institute of Medical Research A Lanari-IDIM, University of Buenos Aires-National Council of Scientific and Technological Research (CONICET), Argentina

## Abstract

**Background:**

Non-alcoholic fatty liver disease (NAFLD) is associated with increased risks of atherosclerotic diseases, including cardiovascular disease. However, the difference in risk between patients with non-alcoholic fatty liver (NAFL) and non-alcoholic steatohepatitis (NASH) has not yet been determined. Accumulating evidence has shown that high amounts of small dense low-density lipoprotein (sdLDL) are closely associated with atherosclerotic diseases. This study investigated differences in risk factors for atherosclerotic diseases, especially LDL-migration index (LDL-MI), an indicator of sdLDL, between patients with NAFL and NASH.

**Methods:**

LDL-MI was analyzed in a primary cohort of 156 patients with NAFLD, including 53 with NAFL and 103 with NASH, and a validation cohort of 69 patients with NAFLD, including 25 with NAFL and 44 with NASH.

**Results:**

In the primary cohort, NASH was associated with elevated LDL-MI (p = 0.039). Multiple regression analysis showed that NASH and the non-use of lipid lowering medications were independently correlated with higher LDL-MI in all patients with NAFLD. Among patients not on lipid lowering medications, those with NASH had significantly higher LDL-MI than those with NAFL (p = 0.001). These findings were confirmed in a validation cohort, in that LDL-MI was significantly higher in patients with NASH than with NAFL (p = 0.043).

**Conclusion:**

This study is the first to show that LDL-MI, an indicator of sdLDL, was higher in patients with NASH than with NAFL, suggesting that the risk of atherosclerotic diseases may be higher in NASH than NAFL. Patients with NASH should be followed closely, especially for the progression of liver pathology and atherosclerotic diseases.

**Trial Registration:**

UMIN000009614

## Background

Non-alcoholic fatty liver disease (NAFLD) is defined by fat accumulation in the liver, exceeding 5% of hepatocytes, in the absence of significant alcohol intake, viral infection, or any other specific etiology of liver disease. NAFLD encompasses a wide spectrum of conditions, ranging from non-alcoholic fatty liver (NAFL) to non-alcoholic steatohepatitis (NASH). About 10–20% of patients diagnosed with NAFLD will eventually exhibit signs of fibrosis, ballooning, necrosis, and inflammation, indicating the development of NASH, a condition that may progress to cirrhosis and end-stage liver disease [1].

The global health risks of NAFLD are not confined to the liver. Studies have shown that patients with NAFLD have increased rates of diabetes mellitus (DM) and atherosclerotic diseases including cardiovascular disease (CVD), as well as of liver-associated complications and mortality, than patients without NAFLD [2]–[3]. Moreover, patients with NAFLD are at higher risk of increased carotid intima media thickness [4]–[5], reduced endothelial function [6], increased coronary artery calcification [7]–[8], and increased arterial stiffness [9]. We previously reported that obesity-induced hyperleptinemia may be associated with NASH through increased chronic systematic inflammation, a risk factor for atherosclerosis, resulting from hyperresponsiveness to gut-derived endotoxins [10]. Endothelial dysfunction has been reported to be worse in patients with NASH than in control and NAFL patients [11].

CVD remains an important cause of death in patients with advanced NASH. For example, a 10-year follow-up showed that CVD accounted for 28% of deaths of patients with NASH-related cirrhosis but only 2% of deaths of patients with hepatitis C virus-related cirrhosis [12]. Despite these findings, it is unclear whether NASH is a marker for atherosclerosis and promotes this disease independently. Moreover, the effects of NASH on the risk of future cardiovascular events have not been well established.

Alterations in lipid metabolism may account for the differential development of NAFL and NASH. We reported that reductions in liver secretion of very low-density lipoprotein (VLDL) in patients with NASH are caused by reductions in VLDL synthesis, with the latter due to the decreased expression of microsomal triglyceride transfer protein (MTTP); moreover, these reductions in VLDL secretion lead to the formation of larger lipid droplets in the livers of these patients [13]–[15]. The surplus triglycerides (TGs) in these livers are therefore transported as TG-rich lipoproteins (VLDL1), a precursor of sdLDL particles [16], which are a stronger risk factor than low-density lipoprotein cholesterol (LDL-C) for atherosclerotic diseases including CVD [17]–[18]. In addition, sdLDL has been associated with several metabolic disorders, including NAFLD [19]–[20], DM [21], hypertension [22], obstructive sleep apnea syndrome [23], and chronic kidney disease [24]. To date, however, no studies have investigated whether sdLDL differs in patients with NAFL and NASH. We hypothesized that risk factors for atherosclerotic diseases, including sdLDL, would be elevated in patients with NASH when compared with healthy subjects and patients with other liver diseases, including NAFL. We therefore assayed serum lipoprotein subclasses and LDL-migration index (LDL-MI), an indicator of sdLDL, in patients with NASH and NAFL.

## Methods

### Subject characteristics

This primary cohort included patients evaluated at two academic medical centers in Japan, Yokohama City University Hospital, Yokohama, and Saga Medical School, Saga, whereas the validation cohort included patients at two other medical centers in Japan, Hiroshima University Hospital, Hiroshima, and Kochi University Hospital, Kochi. The primary cohort consisted of 156 patients and the validation cohort of 69 patients, all of whom had liver biopsy-diagnosed NAFLD. The NAFLD patients enrolled in the validation cohort did not receive lipid lowering medications. Subjects with a history of excessive alcohol consumption (weekly consumption>140 g for men or>70 g for women), other liver diseases such as chronic hepatitis, drug use associated with fatty liver, weight-reduction, renal diseases or thyroid disorders were excluded. The healthy control group consisted of 30 subjects of mean age and sex ratio comparable to those of the NAFL and NASH groups; all had normal liver enzyme levels, and ultrasonography showed no evidence of fatty liver. We also analyzed 141 subjects with other liver diseases, including 32 with alcoholic liver disease (ALD), 38 with chronic hepatitis C (CH-C), 22 with chronic hepatitis B (CH-B), 30 with autoimmune hepatitis (AIH) and 19 with primary biliary cirrhosis (PBC) to determine whether lipid data from NAFLD patients differed significantly from the data of patients with other liver diseases.

The study protocol was reviewed and approved by the ethics review committee of each institution, and all subjects provided written informed consent prior to examination. The study protocol conformed to the ethical guidelines of the Declaration of Helsinki and was conducted with the approval of the Ethics Committee of Yokohama City University Hospital. This trial is registered with the UMIN Clinical Trials Registry as No. UMIN000009614.

### Clinical and laboratory analysis

Blood samples were obtained after an overnight (12 h) fast and at the same time as liver biopsies. Non-HDL-C, which has been shown to be a superior predictor of cardiovascular events [25], was calculated by subtracting high-density lipoprotein cholesterol (HDL-C) from total cholesterol (TC) concentrations. In addition, the LDL-C/HDL-C ratio, a positive predictor of lipid-rich coronary plaques in patients with ischemic heart disease [26], was calculated for each subject.

### Lipoprotein analysis

Blood samples for lipoprotein analysis were obtained from all patients within 6 months of liver biopsy. The diameters of lipoprotein particles were generally determined by polyacrylamide gel electrophoresis (PAGE) using a LipoPhor system (BML, Tokyo, Japan) according to the manufacturer's instructions. This system separates lipoproteins into three major classes: VLDL, LDL and HDL. LDL size was assessed by the migration of the LDL fraction, and LDL-MI calculated as the distance of the LDL peak from the VLDL and HDL peaks [27], [28]. LDL-MI has been reported to show a strong negative correlation with LDL size on PAGE (r = −0.784, p<0.001) [27], with a larger LDL-MI, especially ≥0.35, indicating a higher proportion of sdLDL particles [27], [29]. Actually, we evaluated lipoproteins with high sensitivity using gel-permeation high-performance liquid chromatography (HPLC, LipoSEARCH), which is an alternative method to ultracentrifugation [30], in 49 patients with biopsy-proven NAFLD of primary cohort.

### Histopathologic and immunohistochemical evaluations

Liver biopsy samples were obtained from all patients with NAFLD using an 18 G needle biopsy kit according to a standard protocol; two specimens were obtained from each patient to acquire a sample of sufficient size for analysis and to reduce histological errors. An adequate liver biopsy sample was defined as a>16 mm in length and/or with>10 portal tracts. Livers were assessed histologically by two pathologists as described [31]–[32]. Macrovesicular steatosis affecting at least 5% of hepatocytes was observed in all patients with NAFLD; these patients were classified as having (NASH) or not having (NAFL) steatohepatitis. In addition to steatosis, the minimum criteria for the diagnosis of steatohepatitis included the presence of lobular inflammation, ballooning of cells and perisinusoidal/pericellular fibrosis in zone 3 of the hepatic acini. Steatosis affecting <5%, 5–33%, 33–66%, and>66% of hepatocytes was classified as grades 0–3, respectively. Lobular inflammation was graded according to the number of inflammatory foci per field of view at a magnification of 200×, with 0, <2, 2–4, and>4 foci per field classified as grades 0–3, respectively. Hepatocellular ballooning of no, few and many cells was classified as grades 0–2, respectively. Fibrosis severity was scored as described [31]. Subjects with NASH-associated cirrhosis were defined clinicopathologically [33].

### Lipid lowering medications

NAFLD patients in the primary cohort with dyslipidemia (TG>150 mg/dl and/or LDL-C>140 mg/dl) were prospectively treated, in a single arm, open-label trial, with lipid lowering medications, including 21 patients treated with 10 mg/kg/day ezetimibe; 15 treated with 10 mg/kg/day atorvastatin, an HMG-CoA reductase inhibitor (HMG-CoA RI); and 19 treated with 400 mg/kg/day fibrate for 12 weeks. LDL-MI in these patients was measured before and after treatment with lipid lowering medications.

### Statistical analysis

Continuous variables were summarized as means and standard deviations (SDs); and categorical variables as frequencies and percentages. All statistical analyses were performed using SPSS 12.0 software (SPSS Inc., Chicago, IL, USA). The *t*-test and analysis of variance (ANOVA) with Scheffe's multiple testing correction were used for univariate comparisons between groups. Because many of the variables were not normally distributed, the Kruskal–Wallis test was used for comparisons of more than two independent groups. *P*-values <0.05 were considered statistically significant.

## Results

### Characteristics of the primary cohort

The primary cohort consisted of 156 patients with NAFLD and 30 control subjects. Table 1 (left) shows their principal features and laboratory characteristics. No differences in the prevalence of DM, hypertension, dyslipidemia and obesity were observed between the NAFL and NASH groups. Histological characteristics are also summarized in Table 1 (left). Twenty-three of the 53 patients with NAFL (43.4%) and 60 of the 103 (58.3%) with NASH were treated with lipid lowering therapy; of these 83 patients, 36 (43.4%) were on an HMG-CoA RI, 30 (36.1%) were on ezetimibe and 18 (21.7%) were on fibrate.

**Table 1:** Clinical, serological and histological characteristics of the primary and validation cohorts.

	Primary cohort					Validation cohort		
	Control	NAFL	NASH	P value*	P value**	NAFL	NASH	P value*
Number (n)	30	53	103			25	44	
Age (years)	45.6±12.4	48.6±13.7	50.2±14.7	0.382	0.115	48.2±17.7	48.1±15.0	0.965
Gender (male; female)	18;12	33;20	58; 45	0.344	0.572	15;10	28;16	0.483
Body mass index (kg/m^2^)	22.6±3.2	28.2±5.4	29.3±5.2	0.132	<0.001**	28.7±5.7	28.9±4.8	0.702
AST (IU/l)	25.1±8.1	32.4±10.1	54.3±27.9	0.001*	<0.001**	42.2±36.4	50.2±27.3	0.075
ALT (IU/l)	22.6±7.2	48.7±27.6	78.2±52.2	0.003*	<0.001**	71.2±55.7	86.2±48.9	0.134
C-reactive protein (mg/l)	0.07±0.04	0.12±0.08	0.27±0.43	0.003*	0.002**	0.31±0.58	0.46±1.01	0.108
Creatine (mg/dl)	0.64±0.32	0.75±0.43	0.71±0.55	0.774	0.093	0.73±0.30	0.79±0.41	0.561
Fasting blood glucose (mg/dl)	90.3±15.5	113.2±37.2	114.7±22.9	0.	0.003**	132.5±71.3	117.1±31.2	0.543
Fasting insulin (mU/l)	7.8±5.6	12.1±16.5	16.3±10.0	0.049*	0.002**	17.5±16.5	16.2±7.9	0.318
HbA1c	5.5±0.5	6.1±1.0	6.4±0.9	0.063	0.018**	6.7±1.6	6.4±1.4	0.602
Diabetes mellitus	0	21 (39.6)	54 (52.4)	0.321	<0.001**	12 (48.0)	20 (45.5)	0.421
Hypertension (%)	0	23 (43.3)	42 (40.8)	0.569	<0.001**	9 (36.0)	15 (34.1)	0.787
Dyslipidemia (%)	0	27 (50.9)	64 (62.3)	0.201	<0.001**	14 (56.0)	25 (56.8)	0.656
Lipid lowering therapy (%)	0	23 (43.4)	60 (58.3)	0.099	<0.001**	0	0	
Steatosis grade								
5–33%		21	44			10	19	
33–66%		28	44			13	22	
>66%		4	15			2	3	
Lobular inflammation								
None		7	0			7	0	
<2 foci per 200x field		21	54			14	22	
2–4 foci per 200x field		23	41			4	18	
>4 foci per 200x field		2	8			0	4	
Liver cell ballooning								
None		37	0			34	0	
Few balloon cells		14	74			12	34	
Many balloon cells		2	29			2	10	
Fibrosis stage								
None		25	0			22	0	
Perisinusoidal or periportal		22	57			20	20	
Perisinusoidal and portal/periportal		6	21			6	21	
Bridging fibrosis		0	22			0	3	
Cirrhosis		0	3			0	0	

Numbers represent the mean ± SD. Abbreviations: AST, aspartate aminotransferase; ALT, alanine aminotransferase. P-values* are for comparisons of groups with NAFL and NASH using ANOVA with Scheffe's multiple testing corrections. P-values** are for comparisons among groups of controls, NAFL and NASH using the Kruskal–Wallis test.

### Serum lipid levels and lipoprotein subclasses in the primary cohort

Mean serum TC, LDL-C, TG, and non-HDL-C concentrations and the LDL-C/HDL-C ratio were higher and mean HDL-C concentrations lower in patients with NAFLD, including both NAFL and NASH, than in control subjects [Table 2 (left)]. However, all of these parameters were similar in the NAFL and NASH groups.

**Table 2:** Serum lipid levels and lipoprotein subclasses in the primary and validation cohorts.

	Primary cohort					Validation cohort		
	Control (n = 30)	NAFL (n = 53)	NASH (n = 103)	P value*	P value**	NAFL (n = 25)	NASH (n = 44)	P value*
Serum lipid levels								
Total cholesterol (mg/dl)	168.8±19.8	191.8±23.5	199.5±34.7	0.579	0.007**	197.7±46.4	200.2±35.0	0.753
LDL cholesterol (mg/dl)	102.2±19.4	115.9±18.5	126.2±28.3	0.383	0.032**	126.9±42.4	125.1±32.4	0.769
HDL cholesterol (mg/dl)	59.4±10.2	56.8±9.8	52.8±14.7	0.489	0.041**	54.6±10.8	51.1±12.9	0.167
Total triglycerides (mg/dl)	115.8±64.7	142.1±64.9	158.5±74.4	0.284	0.029**	136.4±53.1	148.4±66.4	0.325
Non-HDL cholesterol (mg/dl)	109.4±24.5	135.0±31.6	146.7±34.5	0.118	0.010**	143.1±38.1	149.1±33.9	0.232
LDL-C/HDL-C	1.7±0.4	2.0±0.6	2.4±0.8	0.244	0.014**	2.3±0.9	2.5±0.9	0.392
Lipoprotein subclasses								
VLDL (%)	13.2±4.5	18.1±4.9	16.0±6.0	0.267	0.039**	15.4±7.0	12.9±7.8	0.092
IDL (%)	3.2±2.8	6.7±8.3	7.8±7.6	0.696	0.019**	6.5±3.2	6.7±2.1	0.725
LDL (%)	45.9±6.9	45.5±8.0	51.5±9.7	0.443	0.483	51.1±9.6	53.8±11.2	0.384
HDL (%)	37.3±12.5	28.8±4.4	26.0±5.7	0.186	0.021**	27.1±5.4	26.6±5.7	0.258
LDL-migration index	0.29±0.03	0.32±0.06	0.37±0.06	0.039*	<0.001**	0.30±0.03	0.34±0.05	0.043*

LDL-migration index, an indicator of sdLDL, was analyzed in patients with NAFLD by PAGE. Numbers represent the mean ± SD. P-values* are for comparisons between NAFL and NASH groups using ANOVA with Scheffe's multiple testing corrections. P-values** are for comparisons among groups of controls, NAFL and NASH using Kruskal–Wallis tests.

**Figure 1:**
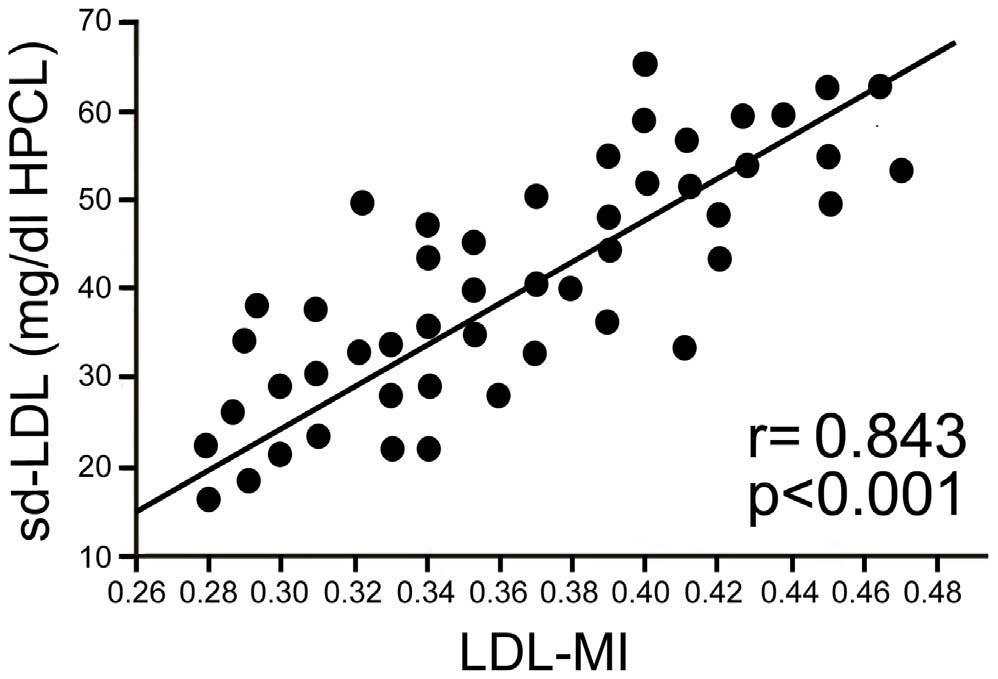
Relationship between LDL-MI, as determined by PAGE, and sdLDL concentrations, measured by HPLC, in 49 patients with NAFLD. LDL-MI was significantly correlated with sdLDL concentrations (r = 0.843, P<0.001).

Results from 49 patients showed that LDL-MI was strongly correlated with sdLDL concentration, as determined by HPLC (Fig. 1). Using PAGE to investigate serum lipoprotein subclasses in patients with NAFLD [27]–[28], we found no differences between the NAFL and NASH groups. However, LDL-MI was significantly higher in the NASH than in either the control or NAFL group [Table 2 (left)].

### Multiple regression analysis of factors associated with increased LDL-MI

To clarify factors associated with increased LDL-MI, factors that may be associated with atherosclerotic diseases were assessed in groups of patients with low (<0.35) and high (≥0.35) LDL-MI (Table 3). Univariate analysis showed that the percentage of NAFLD patients not using lipid lowering medications and the prevalence of NASH were significantly higher (p<0.05 each), and that the prevalence of DM, dyslipidemia, high LDL-C levels, of hypertriglyceridemia tended to be higher (p<0.1 each), in the high than in the low LDL-MI group. Multiple regression analysis that included all of these factors, showed that not using lipid lowering medications and NASH were independent factors associated with increased LDL-MI in patients with NAFLD (Table 4). Indeed, LDL-MI was higher in 50 patients with NASH than in 24 patients with NAFL not on lipid-lowering medications (data not shown). In addition, VLDL concentrations tended to be lower in the NASH than in the NAFL group (p = 0.079, data not shown). In contrast, there was no difference in LDL-MI between the 28 patients with NAFL and the 51 with NASH on lipid-lowering medications (data not shown). Finally, a formal test of interaction of the two major factors associated with LDL-MI, non-use of lipid lowering medications and NASH, showed significant interaction effects (p = 0.006). These results suggest that the risk of atherosclerotic diseases may be higher in patients with NASH than with NAFL not on lipid lowering therapy.

**Table 3:** Clinical characteristics of patients with NAFLD and low or high LDL-MI.

Factors	LDL-MI <0.35 (n = 93)	LDL-MI ≥0.35 (n = 63)	P value*
Age (years)	54.9±13.1	53.2±13.9	0.643
Gender (male; female)	58; 42	33; 23	0.478
Body mass index (kg/m^2^)	27.9±4.0	28.3±5.3	0.598
Renal dysfunction (%)	4.3	3.2	0.781
Diabetes mellitus (%)	43.0	55.5	0.076
Hypertension (%)	39.7	46.0	0.253
Dyslipidemia (%)	51.6	68.3	0.062
High levels of low-density lipoprotein cholesterol (%)	33.3	39.7	0.099
Hypertriglyceridemia (%)	22.6	39.7	0.089
Low levels of high-density lipoprotein cholesterol (%)	16.1	23.8	0.228
Non-use of lipid lowering medications (%)	41.9	69.8	0.008
Nonalcoholic steatohepatitis (%)	54.8	81.0	0.038

Numbers represent the mean ± SD. The prevalence of several diseases was expressed as percentages of whole numbers. P-values are for comparisons between low and high LDL-MI groups using ANOVA with Scheffe's multiple testing corrections.

**Table 4:** Multiple regression analysis of factors associated with increased LDL-MI in patients with NAFLD.

Factors	Regression coefficient	95% CI	P value*
Diabetes mellitus	0.1457	−0.0871–0.3217	0.1706
Dyslipidemia	0.1694	−0.0987–0.3606	0.0976
Hypertriglyceridemia	0.1654	−0.1018–0.3402	0.0892
High levels of low-density lipoprotein cholesterol	0.0520	−0.0761–0.3232	0.2025
Non-use of lipid lowering medications	0.2956	0.0638–0.4672	0.0008*
Nonalcoholic steatohepatitis	0.2522	0.0088–0.4545	0.0429*

The dependent variable was LDL-MI. Independent variables included prevalence of DM, dyslipidemia, high LDL-C concentration, hypertriglyceridemia, non-use of lipid lowering medications, and NASH. CI, confidence interval.

### Effects of lipid lowering therapy on LDL-MI in NAFLD patients

**Figure 2:**
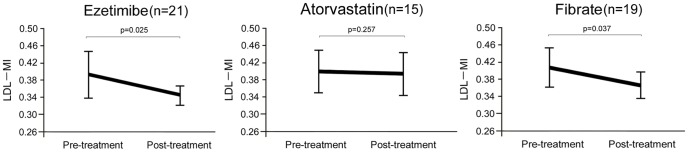
Effects of the lipid lowering drugs (A) ezetimibe, (B) fibrate, and (C) atorvastatin on LDL-MI in patients with NAFLD. All data are expressed as the mean ± SD. Statistical significance was determined using Student's t-test.

Next, we prospectively investigated whether lipid lowering therapies improve LDL-MI in patients with NAFLD. We therefore treated 21, 15 and 19 NAFLD patients with ezetimibe, atorvastatin and fibrate, respectively, for 12 weeks. The characteristics of these patients are shown in Table S1 in S1 File. All three agents improved dyslipidemia (Table S2–4 in S1 File), and ezetimibe and fibrate, but not atorvastatin, significantly reduced LDL-MI (Fig. 2). These results suggest that ezetimibe or fibrate may prevent the onset of atherosclerotic diseases in patients with NAFLD by decreasing LDL-MI, an indicator of sdLDL.

### Characteristics of the validation cohort

The results shown above were validated in an additional 69 patients with biopsy-diagnosed NAFLD not on lipid lowering therapy. Mean age, gender, BMI and the prevalence of DM, hypertension, and dyslipidemia were similar in the NAFL and NASH groups [Table 1 (right)].

### Serum lipid levels and lipoprotein subclasses in the validation cohort

Mean TC, LDL-C, HDL-C, TG, and non-HDL-C concentrations and mean LDL-C/HDL-C ratios were similar in the NAFL and NASH groups. Although the concentrations of serum lipoprotein subclasses were similar in the NAFL and NASH groups, VLDL concentrations tended to be lower in the NASH than in the NAFL group. In addition, LDL-MI was significantly higher in the NASH than in the NAFL group [Table 2 (right)]. These data confirm that elevated LDL-MI is associated with NASH in patients not receiving lipid lowering therapy.

### Disease specificity of LDL-MI for NAFLD

**Figure 3:**
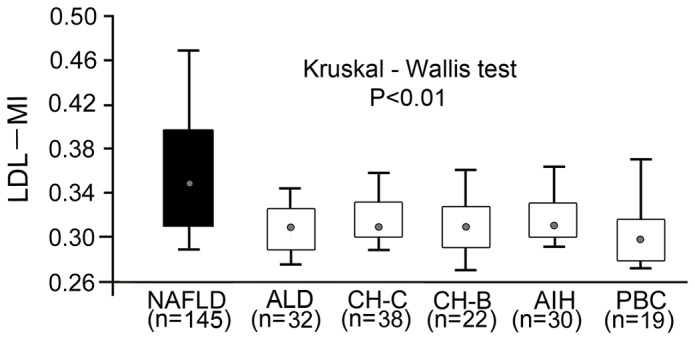
LDL-MI in patients with various liver diseases including NAFLD. LDL-MI was significantly higher in patients with NAFLD patients than in patients with with ALD, CH-C, CH-B, AIH and PBC. Shown are the interquartile ranges (boxes), medians (dots), and ranges (vertical lines).

Finally, we compared LDL-MI across a range of liver diseases, finding it was significantly higher in patients with NAFLD than in patients with other liver diseases and metabolic diseases (p<0.01, Fig. 3). These results clearly indicate that high LDL-MI is specific to NAFLD, suggesting that patients with NAFLD are at higher risk for atherosclerotic diseases, including CVD, than patients with other liver diseases.

## Discussion

This cross-sectional, multicenter study showed that LDL-MI, a surrogate marker of sdLDL, was higher in patients with biopsy-proven NASH than that in patients with NAFL, control subjects and patients with other types of liver disease. In addition, lipid lowering medications, especially ezetimibe and fibrate, decreased LDL-MI in patients with NAFLD. To evaluate whether NASH was associated with elevated LDL-MI in an independent cohort, we evaluated LDL-MI in NAFLD patients from two additional institutions. Despite the limited cohort size, we found that LDL-MI was significantly higher in patients with NASH than with NAFL.

LDL, defined as the lipid fraction of density between 1.019 and 1.063 g/ml on ultracentrifugation, is considered the most atherogenic type of lipoprotein. LDL can be separated into several distinct subclasses differing in size, charge, density, physicochemical composition, and atherogenicity [34]–[36]. LDL subclasses are generated during the delipidation process from very-low-density lipoprotein (VLDL), which yields IDL and LDL particles [34], [36]. In addition to the increase in large buoyant LDL, the predominance of sdLDL has been regarded as a risk factor and surrogate marker for CVD by the National Cholesterol Education Program Adult Treatment Panel III (NCEP ATP III) [37]. sdLDL is regarded as particularly atherogenic, since these particles are retained preferentially by the artery wall, are readily oxidized and carry an enzyme believed to have an important role in atherosclerosis [38].

To our knowledge, this study is the first to assess differences in LDL-MI, an indicator of sdLDL, between patients with NAFL and NASH. We found that LDL-MI was higher in patients with NASH than with NAFL despite the two groups having similar LDL-C concentrations. sdLDL has also been reported closely associated with the prevalence of DM [21], hypertension [22], dyslipidemia [39], obesity [40] and chronic kidney disease [24]. Multivariate regression analysis in patients not taking lipid lowering medications showed that increased LDL-MI was independently associated with NASH. Conversely, NASH was found to be an independent predictor of increased LDL-MI in patients with NAFLD, especially those not on lipid lowering therapy, suggesting a connection with atherosclerotic diseases.

Methodological techniques, including PAGE [41]–[43], ultracentrifugation [44], nuclear magnetic resonance [45], and high-performance liquid chromatography (HPLC) [46], have been used by clinical laboratories to measure the size, number, distribution, cholesterol content, and subclass of LDL particles. This study used PAGE to measure LDL particle size and LDL-MI because of the ability of this method to analyze many samples, as well as its simplicity and accuracy. A study evaluating the relationships between LDL-MI and Rf value, determined by the LipoPhor LDL system, and LDL particle size, measured by non-denaturing 2 to 6% PAGE, found that LDL-MI showed a strong positive correlation with Rf value and a negative correlation with LDL particle size [28]. Results from 49 of our patients showed that LDL-MI was strongly correlated with sdLDL concentration, as determined by HPLC, a method that can measure sdLDL directly but is more expensive and more complicated than PAGE. We therefore used LDL-MI as a measure of sdLDL in patients with NAFLD and other liver diseases, finding that LDL-MI measured by PAGE was significantly predictive of sdLDL (r = 0.843, p<0.001).

**Figure 4:**
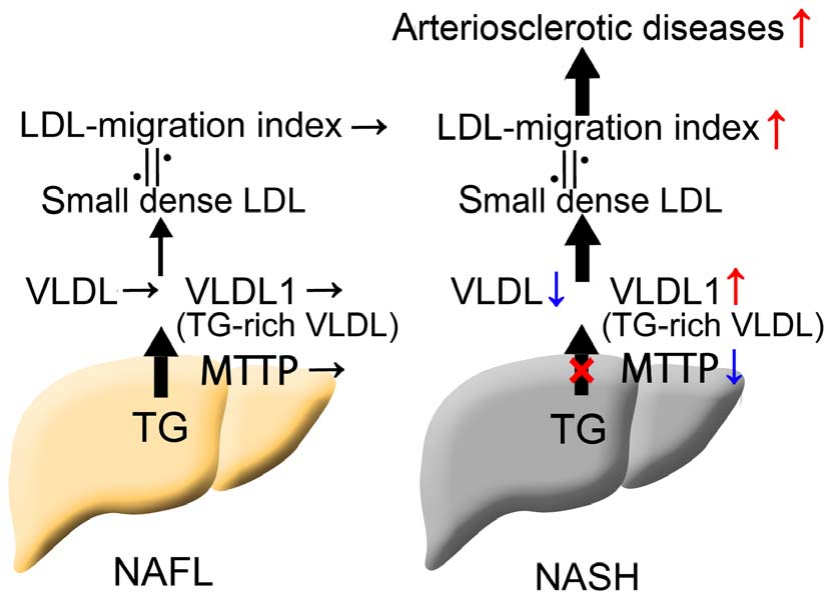
Mechanisms of increased LDL-MI, an indicator of sdLDL, in patients with NASH. Microsomal triglyceride transfer protein activity is much lower in the livers of patients with NASH than with NAFL, resulting in decreased synthesis of total VLDL and increased synthesis of TG-rich VLDL (VLDL1) [13], leading to an increase in sdLDL [16]. The incidence of atherosclerotic diseases may therefore be quite high in patients with NASH.

sdLDL is generated during intravascular lipoprotein remodeling, which results from disturbances in lipid metabolism, such as activation of hepatic lipase (HL), an enzyme that hydrolyzes lipoprotein triglycerides/phospholipids and is associated with the degree of hepatic steatosis [47]. Indeed, we found that LDL-MI correlated significantly with the progression of hepatic steatosis (Fig. S1 in S1 File). However, the degree of hepatic steatosis degree was similar in patients with NAFL and NASH. In addition, the expression of HL mRNA was similar in patients with NASH and NAFL (data not shown). Taken together, these results suggest that mRNA levels of HL were not associated with the higher LDL-MI observed in patients with NASH than in those with NAFL in this study. The key predisposing factor for sdLDL is the development of hypertriglyceridemia, in particular elevated plasma concentrations of large, TG-rich VLDL (VLDL1). We recently reported that VLDL1 was significantly higher in patients with NASH than with NAFL owing to impairments in lipid outflow from the liver resulting from a decrease in microsomal triglyceride transfer protein [13]. Overproduction of VLDL1 leads to the formation of sdLDL [16], resulting in sdLDL being higher in patients with NASH than with NAFL. The proposed mechanisms thought to underlie increased sdLDL in NASH are shown in Fig. 4.

Correction of the hypertriglyceridemic state can reduce the likelihood of sdLDL formation. Peroxisome proliferator-activated receptors (PPARs) in the nucleus are involved in regulating lipid metabolism, with PPAR ligands used to treat dyslipidemia. Fibrate can alter TG metabolism and modulate LDL size and subclasses, including sdLDL. We found that fibrate decreased LDL-MI, an indicator of sdLDL, in NAFLD patients. In addition, fibrate, by reducing sdLDL, has been reported to inhibit atherosclerosis [48]. Fibrate was shown to protect against not only liver steatosis and inflammation but atherosclerosis development in western diet-induced apolipoprotein E2 knock-in mice [49]. Similarly, we found that ezetimibe treatment decreased LDL-MI in patients with NAFLD. Recently, ezetimibe was reported to improve the hypertriglyceridemic state and decrease sdLDL in patients with type 2 DM [50]–[51], as well as to improve major clinical parameters and histological findings in patients with NASH [52]. In contrast, the HMG-CoA RI atorvastatin did not decrease LDL-MI in NAFLD patients. A randomized placebo-controlled trial found that HMG-CoA RIs did not decrease sdLDL in patients with coronary artery disease and were not an effective treatment for NASH [53]–[54]. The ability of HMG-CoA RIs to inhibit atherosclerosis is likely not due to a decrease in sdLDL. These results suggest that fibrate and ezetimibe, but not HMG-CoA RI, can not only improve liver dysfunction but lower the risk of atherosclerotic diseases including CVD by reducing sdLDL in patients with NAFLD.

This study had several limitations. First, we did not directly estimate the proportion of patients with atherosclerotic diseases or CVD events. Second, we did not measure sdLDL concentrations directly, suggesting that further studies are needed to determine the direct associations of LDL-MI and sdLDL with atherosclerosis. Third, the use of liver biopsy as the gold standard for assessing liver pathology has limitations associated with sampling errors as well as intra- and inter-observer variability, which are at least partly linked to the size of the biopsy. Fourth, the patients included in this study were referred from different hepatology centers in Japan with a particular interest in NAFLD; therefore, some referral bias could not be ruled out. Patient selection bias may also have been due to liver biopsies being more likely to be performed on NAFLD patients at risk for NASH.

In conclusion, we found that LDL-MI, a surrogate marker for atherosclerotic diseases, was associated with the occurrence of NASH, especially in patients not on lipid lowering medications. These findings suggested that patients with NASH were at increased risk of atherosclerotic diseases compared with healthy subjects or patients with NAFL. Patients with NASH should be closely monitored for the development of atherosclerotic diseases, including CVD. LDL-MI may be a biomarker for estimating the risk of atherosclerotic diseases in patients with NAFLD.

## Supporting Information

S1 File **Supporting Information File.** Table S1, Clinical, serological and histological characteristics of patients with lipid lowering medications in primary cohort. Table S2, The effect of ezetimibe on serum lipid levels and lipoprotein subclasses in NAFLD patients. Numbers represent the mean ± SD. P-values correspond to the comparison between NAFL and NASH groups using ANOVA with Scheffe's multiple testing corrections. Table S3, The effect of HMG-CoA reductase inhibitors (HMG-CoA RI) on serum lipid levels and lipoprotein subclasses in NAFLD patients. Numbers represent the mean ± SD. P-values correspond to the comparison between patients with NAFL and NASH using ANOVA with Scheffe's multiple testing corrections. Table S4, The effect of fibrate on serum lipid levels and lipoprotein subclasses in NAFLD patients. Numbers represent the mean ± SD. P-values correspond to the comparison between NAFL and NASH using ANOVA with Scheffe's multiple testing corrections. Fig. S1, LDL-MI in the each grade of hepatic steatosis. A steady stepwise increase of LDL-MI is observed with increasing severity of hepatic steatosis degree. The vertical axis represents the LDL-MI and the horizontal axis represents the grade of steatosis. The graph represents the interquartile range (box), median (the dot) and range (lines) of LDL-MI.
(DOC)
