# Supplementary material for: LDL-Migration Index (LDL-MI), an Indicator of Small Dense Low-Density Lipoprotein (sdLDL), Is Higher in Non-Alcoholic Steatohepatitis than in Non-Alcoholic Fatty Liver: A Multicenter Cross-Sectional Study
Source: PLoS One. 2014 Dec 26;9(12):e115403. doi: 10.1371/journal.pone.0115403 (PMC4277307; doi:10.1371/journal.pone.0115403)
Supplement: S1 File — Supporting Information File. Table S1, Clinical, serological and histological characteristics of patients with lipid lowering medications in primary cohort. Table S2, The effect of ezetimibe on serum lipid levels and lipoprotein subclasses in NAFLD patients. Numbers represent the mean ± SD. P-values correspond to the comparison between NAFL and NASH groups using ANOVA with Scheffe's multiple testing corrections. Table S3, The effect of HMG-CoA reductase inhibitors (HMG-CoA RI) on serum lipid levels and lipoprotein subclasses in NAFLD patients. Numbers represent the mean ± SD. P-values correspond to the comparison between patients with NAFL and NASH using ANOVA with Scheffe's multiple testing corrections. Table S4, The effect of fibrate on serum lipid levels and lipoprotein subclasses in NAFLD patients. Numbers represent the mean ± SD. P-values correspond to the comparison between NAFL and NASH using ANOVA with Scheffe's multiple testing corrections. Fig. S1, LDL-MI in the each grade of hepatic steatosis. A steady stepwise increase of LDL-MI is observed with increasing severity of hepatic steatosis degree. The vertical axis represents the LDL-MI and the horizontal axis represents the grade of steatosis. The graph represents the interquartile range (box), median (the dot) and range (lines) of LDL-MI. (DOC) [file pone.0115403.s001.doc]

**Supporting Information**

**Methods**

RNA isolation, reverse transcription and quantification of gene expression using real-time reverse transcription polymerase chain reaction (RT–PCR)

Total RNA was isolated from liver tissue using the RNA Prep mini kit (QIAGEN, Hilden, Germany). Template cDNA synthesis was carried out with 1 mg of total RNA using the High Capacity RNA-to-cDNA kit (Applied Biosystems, Foster City, CA, USA). In a fluorescent temperature cycler, 2.5% of each RT reaction solution was amplified in 25 mL of Fast TaqMan Master Mix (Applied Biosystems) or Fast SYBR Master Mix (Applied Biosystems) containing 0.2 mMof each primer. Samples were incubated in the thermal cycler, with initial denaturation at 95°C for 10 s, followed by 40 cycles under specified conditions. The TaqMan PCR primer used for b-actin gene amplification was 4352935E (Applied Biosystems). Primers of hepatic lipase (HL) used were as follows: forward, 5’-TCAATCATCCGGACACGT TA-3’; reverse, 5’-CTCCCGCGTAAAGGTATGAA-3’.

Table S1.

|  | Ezetimibe | HMG-CoA RI | Fibrate | P value* |
| --- | --- | --- | --- | --- |
| Number (n) | 21 | 15 | 19 |  |
| Age (years) | 46.8±11.2 | 50.2±9.8 | 45.5±13.2 | 0.293 |
| Gender (male; female) | 11;10 | 10;5 | 13; 6 | 0.439 |
| Body mass index (kg/m^2^) | 28.1±4.0 | 27.2±5.1 | 28.4±6.2 | 0.121 |
| AST (IU/l) | 47.4±4.5 | 43.9±10.1 | 48.2±16.4 | 0.271 |
| ALT (IU/l) | 59.3±10.1 | 62.1±19.2 | 56.4±21.5 | 0.451 |
| C-reactive protein (mg/l) | 0.27±0.06 | 0.22±0.09 | 0.19±0.04 | 0.119 |
| Creatine (mg/dl) | 0.81±0.44 | 0.72±0.28 | 0.76±0.33 | 0.681 |
| Fasting blood glucose (mg/dl) | 107.4±18.2 | 111.9±23.1 | 109.3±19.0 | 0.333 |
| Fasting insulin (mU/l) | 14.4±10.1 | 13.2±12.0 | 15.5±10.0 | 0.539 |
| HbA1c | 6.4±0.8 | 6.2±0.5 | 6.9±0.6 | 0.119 |
| Diabetes mellitus | 9 (42.8) | 6 (40.0) | 9 (47.3) | 0.522 |
| Hypertension (%) | 11 (52.4) | 7 (46.7) | 9 (47.3) | 0.215 |
| Steatosis grade |  |  |  |  |
| 5-33% |  | 19 | 41 |  |
| 33-66% |  | 26 | 42 |  |
| >66% |  | 3 | 14 |  |
| Lobular inflammation |  |  |  |  |
| None |  | 6 | 0 |  |
| <2 foci per 200x field |  | 19 | 51 |  |
| 2-4 foci per 200x field |  | 21 | 39 |  |
| >4 foci per 200x field |  | 2 | 7 |  |
| Liver cell ballooning |  |  |  |  |
| None |  | 34 | 0 |  |
| Few balloon cells |  | 12 | 68 |  |
| Many balloon cells |  | 2 | 29 |  |
| Fibrosis stage |  |  |  |  |
| None |  | 22 | 0 |  |
| Perisinusoidal or periportal |  | 20 | 54 |  |
| Perisinusoidal and portal/periportal |  | 6 | 19 |  |
| Bridging fibrosis |  | 0 | 21 |  |
| Cirrhosis |  | 0 | 3 |  |

Table S2.

|  | Before | After | P value* |
| --- | --- | --- | --- |
| Serum lipid levels |  |  |  |
| Total cholesterol (mg/dl) | 204.3±28.3 | 189.2±26.8 | 0.037* |
| LDL cholesterol (mg/dl) | 138.5±21.6 | 119.5±23.9 | 0.041* |
| HDL cholesterol (mg/dl) | 53.9±11.3 | 55.6±13.9 | 0.183 |
| Total triglycerides (mg/dl) | 148.8±37.9 | 125.5±40.9 | 0.049* |
| Non-HDL cholesterol (mg/dl) | 150.4±33.9 | 133.8±31.4 | 0.103 |
| LDL-C/HDL-C | 2.6±0.7 | 2.1±0.7 | 0.129 |
| Lipoprotein subclasses |  |  |  |
| VLDL (%) | 17.2±5.5 | 16.0±5.9 | 0.211 |
| IDL (%) | 6.3±5.9 | 7.6±6.7 | 0.439 |
| LDL (%) | 51.6±9.1 | 48.1±9.9 | 0.131 |
| HDL (%) | 24.9±5.2 | 28.3±7.3 | 0.148 |

Table S3.

|  | Before | After | P value* |
| --- | --- | --- | --- |
| Serum lipid levels |  |  |  |
| Total cholesterol (mg/dl) | 207.7±31.3 | 189.4±28.2 | 0.042* |
| LDL cholesterol (mg/dl) | 140.2±22.9 | 122.7±24.5 | 0.046* |
| HDL cholesterol (mg/dl) | 51.8±10.9 | 54.9±12.2 | 0.293 |
| Total triglycerides (mg/dl) | 150.4±40.2 | 143.8±35.3 | 0.319 |
| Non-HDL cholesterol (mg/dl) | 155.9±36.3 | 134.5±30.7 | 0.097 |
| LDL-C/HDL-C | 2.7±0.9 | 2.2±0.7 | 0.174 |
| Lipoprotein subclasses |  |  |  |
| VLDL (%) | 16.8±4.9 | 17.4±6.9 | 0.272 |
| IDL (%) | 7.4±6.0 | 7.7±6.2 | 0.561 |
| LDL (%) | 52.1±11.4 | 49.9±10.5 | 0.119 |
| HDL (%) | 23.7±5.2 | 25.0±6.9 | 0.186 |

­­­­

Table S4.

|  | Before | After | P value* |
| --- | --- | --- | --- |
| Serum lipid levels |  |  |  |
| Total cholesterol (mg/dl) | 192.6±27.9 | 189.4±29.3 | 0.384 |
| LDL cholesterol (mg/dl) | 134.3±21.5 | 133.9±28.4 | 0.644 |
| HDL cholesterol (mg/dl) | 56.8±12.4 | 60.9±12.1 | 0.382 |
| Total triglycerides (mg/dl) | 163.5±39.5 | 139.4±41.1 | 0.039* |
| Non-HDL cholesterol (mg/dl) | 135.8±38.3 | 128.5±29.4 | 0.539 |
| LDL-C/HDL-C | 2.4±0.7 | 2.2±0.7 | 0.369 |
| Lipoprotein subclasses |  |  |  |
| VLDL (%) | 19.0±7.3 | 17.2±6.3 | 0.114 |
| IDL (%) | 7.8±7.4 | 6.9±6.6 | 0.210 |
| LDL (%) | 47.9±10.1 | 49.4±9.9 | 0.329 |
| HDL (%) | 25.3±5.0 | 26.5±6.2 | 0.199 |

Figure S1.
